# Effects of Infertility Drug Exposure on the Risk of Borderline Ovarian Tumors: A Systematic Review and Meta-Analysis

**DOI:** 10.3390/biomedicines11071835

**Published:** 2023-06-26

**Authors:** Manfei Si, Xiaoxiao Wang, Xueling Song, Xiaoyu Long, Jie Qiao

**Affiliations:** 1 Center for Reproductive Medicine, Department of Obstetrics and Gynecology, Peking University Third Hospital, Beijing 100191, China; 2 National Clinical Research Center for Obstetrics and Gynecology, Peking University Third Hospital, Beijing 100191, China; 3 Key Laboratory of Assisted Reproduction, Peking University, Ministry of Education, Beijing 100191, China; 4 Beijing Key Laboratory of Reproductive Endocrinology and Assisted Reproductive Technology, Beijing 100191, China; 5 Research Center of Clinical Epidemiology, Peking University Third Hospital, Beijing 100191, China; 6 Beijing Advanced Innovation Center for Genomics, Beijing 100191, China; 7 Peking-Tsinghua Center for Life Sciences, Peking University, Beijing 100191, China

**Keywords:** assisted reproduction technology, infertility drugs, IVF, borderline ovarian tumor, meta-analysis

## Abstract

Whether infertility drug exposure increases the risk of borderline ovarian tumors (BOTs) remains controversial. The present study was conducted with a comprehensive search for studies published from January 1990 to December 2021 in the online databases Cochrane Library, PubMed, Web of Science and EMBASE. We considered the first diagnosis of a BOT as the primary outcome. The odds ratio (OR) was calculated with corresponding 95% confidence intervals (CIs) for the risk of BOTs in patients who were treated with infertility drugs. Ten studies, a total of 2,779,511 women, qualified for inclusion in this meta-analysis. The pooled OR of 1.56 (95% CI: 1.09–2.22) revealed a significant positive association between infertility drugs and an increased risk for BOTs, but for specific drugs, only CC plus Gn had statistical significance. No publication bias was detected using the Egger and Begg tests (*p* > 0.05). A significant difference in BOT incidence was observed among infertile women and nulliparous women who were treated with or without infertility drugs. In conclusion, the use of infertility drugs may increase the risk of BOTs, but a dose-dependent relationship was not observed between the number of assisted reproduction technology cycles and the risk of BOTs, and infertile women who successfully became pregnant might have a reduced risk. Registration: PROSPERO, CRD42022330775.

## 1. Introduction

The global incidence of infertility is increasing annually due to environmental pollution, mental and psychological pressure, delayed childbearing and other factors [1,2,3]. A variety of conditions, including ovulation disorders, tubal factors, male factors and unexplained factors, lead to infertility [4]. Assisted reproductive technologies (ARTs) are widely applied as effective treatments for infertility. ART involves the use of infertility drugs that promote the maturation of multiple follicles and subsequently induce multiple ovulations. Commonly used fertility drugs include (1) ovulation-inducing drugs such as clomiphene citrate (CC) and letrozole and (2) controlled ovarian stimulation drugs such as gonadotrophin (Gn), human menopausal gonadotrophin (hMG), human chorionic gonadotrophin (hCG) and progesterone. These drugs are used either alone or in combination according to an ovulation stimulation protocol.

Exposure to these hormones raises concerns about the long-term safety of ovarian stimulation, such as ovarian malignancies [5,6,7]. The common hypotheses are described as high doses of Gns and increased levels of estrogen may stimulate the ovarian surface epithelium and promote the development of ovarian tumors, and multiple ovulations increase mechanical trauma and the susceptibility to DNA damage and malignant transformation (genetic alterations) of the ovarian surface epithelium [8]. A number of studies have been conducted to evaluate the relationship between infertility drugs and the risk of ovarian tumors. Most recent studies have not reported a statistically significant association between invasive ovarian cancer and the use of infertility drugs [9]. The latest results from a large, nationwide cohort study by Spaan et al. published in 2021 consistently showed that ART-treated women did not have an increased risk of ovarian cancer compared with infertile women who were not treated with ART. New theories that epithelial ovarian cancer originates from the fallopian tubes but not from the ovary itself have been proposed in recent years; this finding may refute incessant ovulation and explain this result [10].

However, for borderline ovarian tumors (BOTs), the results are more controversial. BOTs, also known as low malignant potential tumors, account for 15–20% of epithelial ovarian tumors. The pathological characteristic of BOTs is the dysplasia of ovarian epithelial cells without frank invasion by the tumor nodules microscopically. BOTs have been proposed to be separate entities with a different etiology from ovarian cancer [11]. The association with infertility drugs was difficult to determine, especially due to the low event rates of BOTs and multitude of possible confounding factors, such as infertility itself and parity. Neither Asante et al. nor Bjørnholt et al. observed a significant association between the use of infertility drugs and the risk of BOTs [12,13]. However, Reigstad et al. and Spaan et al. found that ART exposure increased the risk of BOTs, but no dose-response relationship with ART cycles was observed [14,15]. Several meta-analyses have investigated the risk of BOTs in relation to ART. Rizzuto et al. concluded that exposure to infertility drugs may increase the risk of BOT, but a meta-analysis was not conducted [16]. Barcroft et al. did not observe a significant difference in ovarian cancer between the fertility treatment (FT) and no-FT groups, but a significant association with BOTs was noted (OR = 1.32, 1.27–2.25); however, the authors did not include studies that Stewart et al. and Bjørnholt et al. published on the same topic [11,13] and the most recent large cohort study conducted by Spaan et al. [14]. Moreover, potential confounders, including infertility, parity, and a cause of infertility, were not considered separately to analyze the relationship. Therefore, conducting an updated systematic review including all available evidence is important. The present systematic review and meta-analysis aimed to provide updates on the effect of the use of infertility drugs during ART on the risk of BOTs.

## 2. Materials and Methods

### 2.1. Literature Search

This review was conducted according to the Preferred Reporting Items for Systematic Reviews and Meta-Analyses (PRISMA) guidelines [17]. The protocol of this present review was registered in PROSPERO (International Prospective Register of Systematic Reviews) (CRD42022330775). We conducted a comprehensive search for studies published from January 1990 to December 2021 in the online databases Cochrane Library, PubMed, Web of Science, and EMBASE. The aim of the search was to identify the risk of BOTs in patients who received infertility drug treatment during ART; thus, a full spectrum of terms encompassing infertility drugs and BOTs were included. The MeSH terms used within the search included ‘infertility drugs’ or ‘exposure to infertility drugs’ or ‘infertility agents’ or ‘use of infertility drugs’ or ‘infertility drug use’ or ‘fertility drugs’ or ‘fertility agents’ or ‘ovulation-stimulating drugs’ or ‘ovarian stimulating drugs’ or ‘ovarian stimulation’ or ‘ovulation stimulation’ or ‘ovulation induction’ or ‘follicular stimulation’ or ‘ovulation-inducing drugs’ or ‘clomiphene citrate’ or ‘gonadotrophin’ or ‘gonadotrophin-releasing hormone analogue’ or ‘gonadotrophin-releasing hormone agonist’ or ‘gonadotrophin-releasing hormone antagonist’ or ‘human chorionic gonadotropin’ or ‘human menopausal gonadotropin’ or ‘LH/hCG action’ or ‘progesterone’ or ‘hormonal infertility treatment’ or ‘use of fertility medication’ or ‘infertility treatment’ or ‘assisted reproductive technology’ or ‘in vitro fertilization’ or ‘controlled ovarian hyperstimulation’ or ‘reproductive techniques’ AND ‘borderline ovarian tumor’ or ‘ovarian tumors of low malignant potential’ or ‘borderline ovarian malignancy’ or ‘borderline ovarian neoplasms’ or ‘ovarian tumors of borderline malignancy’ or ‘borderline malignancy of the ovary’. We also checked the cited references in these primary articles and reviews to ensure that all relevant studies were identified.

### 2.2. Inclusion Criteria and Study Selection

This systematic review and meta-analysis assessed the use of infertility drugs during ART on the risks of BOTs. All studies (case–control studies and cohort studies) written in English reporting the incidence of BOTs in both the treatment group (infertility drugs) and the control group were eligible for inclusion. Some studies pooled the patients exposed to ART, and these were screened and included if data on infertility drug use were available in the article.

Duplicates were removed. Reviews were excluded. Studies that did not report separate data for BOTs were excluded. Studies that only reported combined data for all types of ART were excluded.

### 2.3. Data Extraction and Outcomes

Two authors independently screened all the studies and extracted data. Disputes were resolved through discussion. If no consensus was reached, the third senior author helped make a final decision. The study quality of the cohort and case–control studies was assessed using the Newcastle–Ottawa Scale (NOS) scores based on the quality of the selection, comparability, and outcome/exposure (a total of 8 items in 3 categories) [18]. High-quality choices were identified with a star. A study was awarded a maximum of one star for each item within the “Selection” and “Exposure/Outcome” categories; a maximum of two stars was allowed for “Comparability”. For case–control studies, the maximum score was 9 stars, and for cohort studies, the maximum score was 13 stars. Studies with a score of 6 or more were considered good-quality studies.

### 2.4. Statistical Analysis

The meta-analysis was performed using Stata software version 12.0. Study results were compared by calculating ORs with corresponding 95% confidence intervals (CIs). Heterogeneity between studies was evaluated using the Cochran’s Q and I-squared statistic (I^2^), in which proportions of 25%, 50%, and 75% indicated low, moderate, and high heterogeneity, respectively [19]. The combined risk estimates were computed using a fixed-effects model or, in the presence of heterogeneity (I^2^ was more than 50%), a random-effects model. When the effect models were contradicting in the different subgroup analysis, the random-effects model was usually chosen to be combined to obtain a more reliable result. Forest plots were created to visualize ORs. Publication bias was evaluated by constructing funnel plots, and a sensitivity analysis was performed to assess the effect (leverage) of each study on the results. A *p* value less than 0.05 was considered statistically significant.

## 3. Results

### 3.1. Study Selection

**Figure 1:**
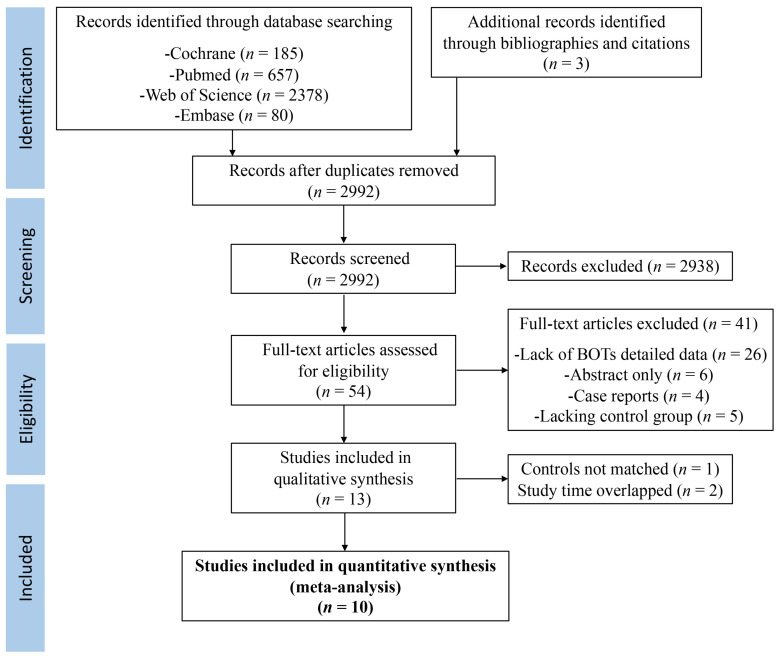
A flowchart of the selection process.

The literature search yielded 3300 studies, of which 12 were included that met the predefined inclusion and exclusion criteria for further consideration [11,13,14,15,20,21,22,23,24,25,26,27]. In view of a significant time overlap in the same area of Denmark [13,21] and the Netherlands [14,24], the studies of Bjornholt et al. and Spaan et al. with a longer time span were finally selected to be involved in the present meta-analysis to avoid double-counting certain patients and cases. A flowchart of the selection process is shown in Figure 1.

### 3.2. Characteristics of the Included Studies

Overall, 10 studies analyzing 2,779,511 patients were included. Among these studies, three were case–control studies, one was a case–cohort study, and the others were cohort studies. Table 1 summarizes the detailed characteristics of the included studies. Quality assessments with the NOS scores of each study are shown in Supplementary Tables S1 and S2.

### 3.3. Meta-Analysis (Outcomes)

**Figure 2:**
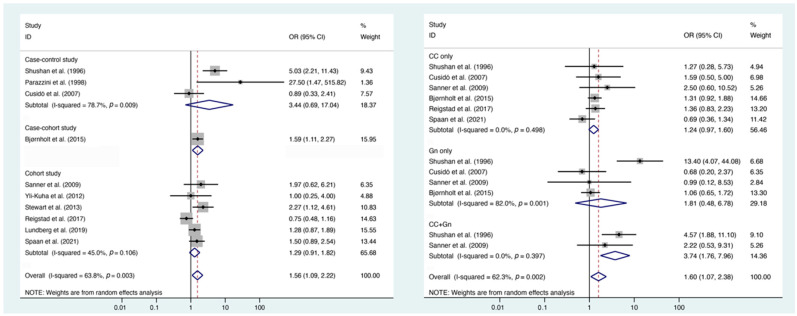
Risk of BOTs in women treated with or without infertility drugs. (**a**) Risk of BOTs in women treated with or without infertility drugs; (**b**) Risk of BOTs in women treated with or without specific infertility drugs [11,13,14,15,20,22,23,25,26,27].

An analysis was conducted using the available information from the 10 studies to investigate the risk of BOTs in patients who were treated with infertility drugs. As shown in Figure 2a, a substantial heterogeneity was observed among the studies (I^2^ = 63.8%, *p* = 0.003); thus, the random effects model was used. The pooled OR was 1.56 (95% CI: 1.09–2.22). Overall, a significant difference in the incidence of BOTs was observed, indicating that patients exposed to any infertility drugs had an increased risk of BOTs. As for the specific drug used, the results were as follows (Figure 2b): CC: OR, 1.24 (95% CI: 0.97–1.60), I^2^ = 0; Gn: OR, 1.81 (95% CI: 0.48–6.78), I^2^ = 82%; and CC + Gn: OR, 3.74 (95% CI: 1.76–7.96), I^2^ = 0. Data from women treated with CC combined with Gn were statistically significant, while the use of CC or Gn alone was not associated with a significant difference in BOT incidence between women with or without infertility drug use. The detailed counts for exposure and controls are shown in Supplementary Table S3.

### 3.4. Publication Bias and Sensitivity Analysis

**Figure 3:**
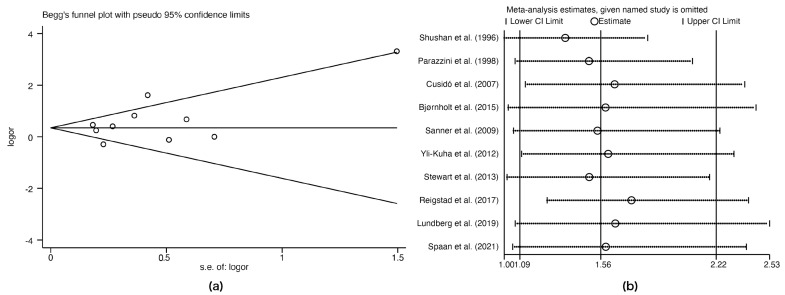
Tests for publication bias and the sensitivity analysis. (**a**) Publication bias; (**b**) Sensitivity analysis [11,13,14,15,20,22,23,25,26,27].

Given the substantial heterogeneity among the included studies, we assessed publication bias and performed a sensitivity analysis. As shown in Figure 3a, the studies were symmetrically distributed in the funnel plot, indicating that no publication bias existed (Egger: *p* = 0.669). The sensitivity analysis revealed that the removal of each study did not alter the final result, indicating that the main result was reliable (Figure 3b).

### 3.5. Subgroup Analysis

Next, we performed a subgroup analysis to assess the contributions of relevant risk factors, including the infertility diagnosis and parous status, to the incidence of BOTs. The detailed counts for exposure and controls are shown in Supplementary Table S3.

#### 3.5.1. Infertility Diagnosis

The five cohort studies included 140,576 women with an infertility diagnosis. Using this population, we found that infertility drugs may increase the risk of BOTs in infertile women treated with infertility drugs, with an OR of 1.59 (95% CI: 1.26–2.00), compared to infertile women not treated (Supplementary Figure S1).

#### 3.5.2. Parous Status

Regarding the effect of parity on the risk of BOTs, we stratified the population into parous women and nulliparous women. As shown in Supplementary Figure S2, for nulliparous women, the use of infertility drugs significantly increased the BOT risk, with an OR of 1.79 (95% CI: 1.25–2.56). However, the differences in the BOT incidence among parous women with or without the use of infertility drugs were not statistically significant: OR, 1.11 (95% CI: 0.60–2.03) (Supplementary Figure S3).

## 4. Discussion

Recently, the relationship between the use of infertility drugs and the risk of BOTs has attracted increasing attention as the application of ART has become more prevalent. One view is that ovulation-stimulating drugs may increase the potential risk of BOTs because repeated ovulation and oocyte retrieval may increase the mechanical trauma and microenvironment with supraphysiological doses of estrogen levels, which produce excessive proliferation and a higher risk of malignant transformation of ovarian epithelial cells. Another view is the opposite, which is based on epidemiological data suggesting no significant correlation between ovulation-stimulating drugs and the occurrence of BOTs. By performing in vitro cell experiments, some scholars also found that BOT cells do not exhibit an obvious proliferative response in the superovulation environment stimulated by FSH and E_2_, and hCG might even significantly inhibit the proliferation of BOT cells. Facing the two contradictory views mentioned above, this study aimed to systematically elucidate the association between ovulation-stimulating drugs and the risk of BOTs.

Overall, this meta-analysis included 10 studies that assessed 2,779,511 patients, and the results suggest that treatment with any infertility drugs was associated with a significantly increased risk of BOTs, OR, 1.56 (95% CI: 1.09–2.22), compared to women who did not use infertility drugs. After examining drug-specific outcomes, CC + Gn exposure (OR, 3.74; 95% CI: 1.76–7.96) was significantly associated with a higher incidence of BOTs. Conversely, the remaining associations between individual infertility drugs (CC and Gn) and BOT incidence were not statistically significant. No publication bias was observed in this study, and a sensitivity analysis was performed and showed no difference in the final result after removing each study. The subgroup analysis showed a higher pooled OR of 1.59 (95% CI: 1.26–2.00) among infertile women. In addition, infertility drugs were associated with a significantly higher BOT incidence among nulliparous women, OR, 1.79 (95% CI: 1.25–2.56), while among parous women, the difference was not statistically significant.

BOTs are rare tumors with an incidence of 4.5/100,000 cases [28]. In contrast to ovarian cancer, they are generally indolent tumors. Women with BOTs tend to be younger (less than 40 years old) and are candidates for fertility-sparing surgery with a relatively good prognosis [29,30]. Based on the present pooled OR value of 1.56 (1.09, 2.22), the use of any infertility drugs may be associated with the increased incidence of BOTs. However, it is uncertain whether patients desiring infertility care would ever be dissuaded from pursuing treatment based on this concern of a typically nonlethal event. Patients should still be clearly informed that although a BOT is not a lethal disease, further surgery and the malignant potential must be considered. Thus, an understanding of the relationship between the use of infertility drugs and BOT risk is important for women considering starting ART treatment as well as clinicians.

Earlier studies reported a greater risk of BOTs due to CC exposure [23,31]. However, many recent studies have not observed an association [13,15,24]. In our study, we also failed to identify a significant difference in the risk of BOTs (1.24, 95% CI: 0.97–1.60) among women exposed to CC. In addition, no significant associations between Gn and BOT incidence were found in the present study, which is consistent with previous studies [13,21,22,23]. Although this present meta-analysis found the use of any infertility drugs increased the risk of BOTs, for specific drugs, only CC plus Gn had statistical significance. This finding still needs to be investigated further with more data, and the effects of additional confounders, such as the age, cause of infertility, endometriosis, and dosage of infertility drugs, should be considered [6,32,33].Our results are consistent with previous studies published by Harris et al. and Barcroft et al., who both noted an increased risk of BOT incidence in women exposed to fertility drugs [34,35]. It should be pointed out that infertility itself is a risk factor for developing ovarian neoplasia and, when controlled for, may account for the increase [36]. Our subgroup analysis showed that among infertile women, the pooled OR value was 1.59 (95% CI: 1.26–2.00). This value was higher than that obtained among all women, which suggests that infertility increased the risk of BOTs. Rossing et al. reported a substantially higher risk of BOTs than expected based on rates in the general population of women, with an age-standardized incidence ratio (SIR) of 3.3 (95% CI: 1.1–7.8) [31]. van Leeuwen et al. reported an increased risk of BOTs compared to the general population, with an SIR of 1.59 (95% CI: 1.1–2.2) [24]. A study conducted by Williams et al. included data for a large population of 255,786 women, and an increased risk of BOTs, with an SIR of 1.36 (95% CI: 1.15–1.6) and an average of 1.7 cases per 100,000 people per year, was detected [6]. The aforementioned results suggest that infertility itself is associated with a higher BOT risk, regardless of whether the use of infertility drugs increases the risk.

The parity status is also an important risk factor for BOTs. Similar to other researchers [14,24], we observed that infertility drug exposure was associated with a significantly higher BOT incidence among nulliparous women, with an OR of 1.79 (95% CI: 1.25–2.56). Interestingly, among parous women, no significant difference in the risk of BOTs was observed between the two groups, with an OR of 1.11 (95% CI: 0.60–2.03). As stated in previous studies, pregnancy is presumed to be a protective factor against BOTs [37,38]. Combined with the previous subgroup analysis of infertility, we suggest that a good pregnancy outcome in infertile women may reduce the BOT risk. As reported by Spaan et al. in 2021, among infertile women treated with ART compared with non-ART treatment, a statistically significant adjusted hazard ratio (HR) was observed in nulliparous women (5.54, 95% CI: 1.33–22.99) but not in parous women (1.19, 95% CI: 0.66–2.16) [14]. Further studies are needed to confirm whether parity might counteract the increased risk associated with the use of infertility drugs.

BOTs are mainly classified as serous, mucinous, and other rare types. Serous BOTs are the most common subtype, accounting for 50–60% of BOTs [39]. A recent study from Spaan et al. found a significantly increased risk of serous borderline ovarian tumors among ART-treated women, but not nonserous borderline tumors [14]. Bjørnholt et al. analyzed the risk for serous and mucinous BOTs separately in their study. They found that mucinous BOTs were not significantly associated with the use of any infertility drugs or specific infertility drugs, while the risk for serous BOTs was increased with any use of progesterone (RR, 1.82; 95% CI: 1.03–3.24) and a long follow up (4–7 years) [13]. These findings offered evidence on the association between infertility drugs used and the specific subtypes of BOTs. However, the present study did not distinguish the subtypes of BOTs due to a lack of adequate information. Thus, further studies should focus on the risk of different subtypes of BOTs.

In addition, we concluded the association of the dose and IVF cycles with the risk of BOTs (Table 2). The present data (including SIR and HR) do not appear to show a dose-dependent relationship; namely, the BOT risk did not increase with the number of ART cycles. Theoretically, if ART treatment increases exposure to hormones and the damage and malignant transformation of epithelial cells, thus increasing the risk of BOTs, a higher BOT risk with more ART cycles should be observed. This lack of a dose-dependent result did not support a causal association and posed certain challenges to our conclusions. Larger prospective cohort studies with a prolonged follow up are needed to analyze the BOT risk and the use of infertility drugs.

In this meta-analysis, substantial heterogeneity was observed among these studies. The possible explanations may be the long-time span of the included studies and differences in the sample size and data sources among the studies. Few studies contained and controlled for the infertility diagnosis, family history of ovarian cancer, and additional comorbidities such as endometriosis. The lack of a follow up varied greatly between studies. Given an earlier presentation age, more cases of BOTs than invasive ovarian cancer would likely be detected within a relatively short follow up. In addition, a greater chance of detecting BOTs in infertile women receiving medical monitoring and treatment has been noted. Previous studies had methodological limitations, including selection bias, a small sample size, a short follow up, and a lack of consideration of potentially important confounders such as infertility and the parity status, the underlying causes of infertility, and various types and cycles of infertility drugs. Therefore, the conclusions listed above must be verified by more high-quality and large-scale clinical studies.

## 5. Conclusions

In summary, the present study showed that the use of infertility drugs appeared to be positively associated with an increased risk of BOTs (OR = 1.56, 95% CI: 1.09–2.22), and the separate analysis of specific drugs showed only CC plus Gn revealed a significant association (OR = 3.74, 95% CI: 1.76–7.96). However, this finding must be verified because a dose-dependent relationship was not observed. Meanwhile, previous studies with methodological limitations and a lack of adjustment for important confounding factors made the results further debatable. In addition, as pointed out in many previous studies, the small number of cases makes it difficult to determine whether it is the diagnosis of infertility or the treatment of infertility drugs that results in the increase; thus, the association does not imply causation. Additionally, parity might further reduce this risk. The information from this meta-analysis might be valuable to both clinicians and patients in their decision making. Certainly, further large-scale studies that consider other confounding factors, such as parity, genetic factors, and the etiology of infertility, and include longer follow-up times are needed.

## Figures and Tables

**Table 1:** Characteristics of the included studies.

Author, Year	Study Period	Country	Study Design	BOT Cases Exposed to Infertility Drug Use	Total no. of Infertility Drug Uses	BOT Cases Not Exposed to Infertility Drug Use	Total no. of Non-Infertility Drug Uses	Median Follow-Up Time (Years)
Shushan et al., 1996 [20]	1990–1993	Israel	Case–control	10	39	26	405	NA
Parazzini et al., 1998 [27]	1986–1991	Italy	Case–control	4	4	89	362	NA
Cusidó et al., 2007 [22]	1982–2000	Spain	Case–control	5	39	37	260	NA
Sanner et al., 2009 [23]	1961–1975	Sweden	Cohort	7	1153	5	1615	33 (1–47)
Yli–Kuha et al., 2012 [25]	1996–1998	Finland	Cohort	4	9175	4	9175	7.75
Stewart et al., 2013 [11]	1982–2002	Western Australia	Cohort	17	7544	14	14,095	16.9
Bjørnholt et al., 2015 [13]	1963–2006	Denmark	Case–cohort	89	772	53	698	11.3
Reigstad et al., 2017 [15]	2004–2014	Norway	Cohort	20	56,194	619	1,297,530	11
Lundberg et al., 2019 [26]	1982–2012	Sweden	Cohort	27	38,003	720	1,301,911	9.5–14.6
Spaan et al., 2021 [14]	1983–2000	Netherlands	Cohort	79	30,565	17	9972	24

**Table 2:** Risk of BOTs associated with the number of ART cycles.

Studies	Different Effect Sizes with 95% CI		
van Leeuwen et al., 2011 [24]	No. of cycles	SIR	95% CI
	1–2 cycles	1.70	0.97–3.74
	3–4 cycles	1.99	1.22–4.14
	≥5 cycles	1.45	0.47–3.38
Spaan et al., 2021 [14]	No. of cycles	SIR	95% CI
	1–2 cycles	2.07	1.28–3.16
	3–4 cycles	2.38	1.51–3.58
	5–6 cycles	1.70	0.62–3.69
	≥7 cycles	2.9	1.07–6.32
Williams et al., 2018 [6]	No. of cycles	SIR	95% CI
	1	1.39	1.08–1.77
	2	1.40	0.95–1.98
	3–4	0.75	0.37–1.33
Bjørnholt et al., 2015 [13]	No. of cycles	Adjusted RR ^a^	95% CI
All BOTs	1–3 cycles	1.12	0.70–1.80
	≥4 cycles	0.91	0.57–1.45
For serous BOTs	1–3 cycles	1.21	0.70–2.08
	≥4 cycles	1.02	0.59–1.74
For mucinous BOTs	1–3 cycles	0.78	0.28–2.20
	≥4 cycles	0.68	0.27–1.72
Spaan et al., 2021 [14]	No. of cycles	Adjusted HR ^b^	95% CI
	1–2 cycles	1.84	1–3.37
	3–4 cycles	2.04	1.12–3.71
	≥5 cycles	1.55	0.78–3.09
Reigstad et al., 2017 [15]	No. of cycles	Adjusted HR ^c^	95% CI
For nulliparous women	1 cycle	1.55	0.47–5.10
	2 cycles	3.25	1.12–9.38
	≥3 cycles	0.62	0.08–4.66
For parous women	1 cycle	1.52	0.55–4.23
	2 cycles	2.72	0.97–7.59
	≥3 cycles	2.59	0.92–7.32

Abbreviations: BOTs, borderline ovarian tumors; ART, assisted reproductive technology; CI, confidence interval; SIR, stasndardized incidence ratio; RR, relative risk; HR, hazard ratio. Note: ^a^ Adjusted for parity status (nulliparous/parous). ^b^ Adjusted for age at start treatment or first visit to gynecologist, parity, and tubal subfertility. ^c^ Adjusted for region of residence, birth cohort, and concomitant exposure to ART.

## Data Availability

The data presented in this study are available in the article and in its Supplementary Materials.

## Supplementary Materials

The following supporting information can be downloaded at: https://www.mdpi.com/article/10.3390/biomedicines11071835/s1, Figure S1: Risk of BOTs among infertile women stratified according to the use of infertility drugs; Figure S2: Risk of BOTs among nulliparous women stratified according to the use of infertility drugs; Figure S3: Risk of BOTs among parous women stratified according to the use of infertility drugs; Table S1: Newcastle–Ottawa Scale for assessment of quality of included studies—Case–control studies; Table S2: Newcastle–Ottawa Scale for assessment of quality of included studies—Cohort studies; Table S3: Counts for BOT events and total events for exposures and controls. References [11,13,14,15,20,22,23,25,26,27] are cited in Supplementary Files.

## References

[1] Carre J., Gatimel N., Moreau J., Parinaud J., Leandri R. (2017). Does air pollution play a role in infertility? A systematic review. Env. Health.

[2] Balasch J., Gratacos E. (2012). Delayed childbearing: Effects on fertility and the outcome of pregnancy. Curr. Opin. Obs. Gynecol..

[3] Kearney A.L., White K.M. (2016). Examining the psychosocial determinants of women’s decisions to delay childbearing. Hum. Reprod..

[4] Carson S.A., Kallen A.N. (2021). Diagnosis and Management of Infertility: A Review. JAMA.

[5] Whittemore A.S., Harris R., Itnyre J., Halpern J. (1992). Characteristics relating to ovarian cancer risk: Collaborative analysis of 12 US case-control studies. I. Methods. Collaborative Ovarian Cancer Group. Am. J. Epidemiol..

[6] Williams C.L., Jones M.E., Swerdlow A.J., Botting B.J., Davies M.C., Jacobs I., Bunch K.J., Murphy M.F.G., Sutcliffe A.G. (2018). Risks of ovarian, breast, and corpus uteri cancer in women treated with assisted reproductive technology in Great Britain, 1991–2010: Data linkage study including 2.2 million person years of observation. BMJ.

[7] Vassard D., Schmidt L., Glazer C.H., Lyng Forman J., Kamper-Jorgensen M., Pinborg A. (2019). Assisted reproductive technology treatment and risk of ovarian cancer-a nationwide population-based cohort study. Hum. Reprod..

[8] Beral V., Doll R., Hermon C., Peto R., Reeves G., Collaborative Group on Epidemiological Studies of Ovarian Cancer (2008). Ovarian cancer and oral contraceptives: Collaborative reanalysis of data from 45 epidemiological studies including 23,257 women with ovarian cancer and 87,303 controls. Lancet.

[9] Jensen A., Sharif H., Frederiksen K., Kjaer S.K. (2009). Use of fertility drugs and risk of ovarian cancer: Danish Population Based Cohort Study. BMJ.

[10] Kurman R.J., Shih Ie M. (2010). The origin and pathogenesis of epithelial ovarian cancer: A proposed unifying theory. Am. J. Surg. Pathol..

[11] Stewart L.M., Holman C.D., Finn J.C., Preen D.B., Hart R. (2013). In vitro fertilization is associated with an increased risk of borderline ovarian tumours. Gynecol. Oncol..

[12] Asante A., Leonard P.H., Weaver A.L., Goode E.L., Jensen J.R., Stewart E.A., Coddington C.C. (2013). Fertility drug use and the risk of ovarian tumors in infertile women: A case-control study. Fertil. Steril..

[13] Bjornholt S.M., Kjaer S.K., Nielsen T.S., Jensen A. (2015). Risk for borderline ovarian tumours after exposure to fertility drugs: Results of a population-based cohort study. Hum. Reprod..

[14] Spaan M., van den Belt-Dusebout A.W., Lambalk C.B., van Boven H.H., Schats R., Kortman M., Broekmans F.J.M., Laven J.S.E., van Santbrink E.J.P., Braat D.D.M. (2021). Long-Term Risk of Ovarian Cancer and Borderline Tumors After Assisted Reproductive Technology. J. Natl. Cancer Inst..

[15] Reigstad M.M., Storeng R., Myklebust T.A., Oldereid N.B., Omland A.K., Robsahm T.E., Brinton L.A., Vangen S., Furu K., Larsen I.K. (2017). Cancer Risk in Women Treated with Fertility Drugs According to Parity Status-A Registry-based Cohort Study. Cancer Epidemiol. Biomark. Prev..

[16] Rizzuto I., Behrens R.F., Smith L.A. (2019). Risk of ovarian cancer in women treated with ovarian stimulating drugs for infertility. Cochrane Database Syst. Rev..

[17] Shamseer L., Moher D., Clarke M., Ghersi D., Liberati A., Petticrew M., Shekelle P., Stewart L.A., Group P.-P. (2015). Preferred reporting items for systematic review and meta-analysis protocols (PRISMA-P) 2015: Elaboration and explanation. BMJ.

[18] Wells G.A., Shea B., O’Connell D., Peterson J., Welch V., Losos M., Tugwell P. (2009). The Newcastle-Ottawa Scale (NOS) for Assessing the Quality if Nonrandomized Studies in Meta-Analyses. http://www.ohri.ca/programs/clinical_epidemiology/oxford.htm.

[19] Higgins J.P., Thompson S.G., Deeks J.J., Altman D.G. (2003). Measuring inconsistency in meta-analyses. BMJ.

[20] Shushan A., Paltiel O., Iscovich J., Elchalal U., Peretz T., Schenker J.G. (1996). Human menopausal gonadotropin and the risk of epithelial ovarian cancer. Fertil. Steril..

[21] Mosgaard B.J., Lidegaard O., Kjaer S.K., Schou G., Andersen A.N. (1998). Ovarian stimulation and borderline ovarian tumors: A case-control study. Fertil. Steril..

[22] Cusido M., Fabregas R., Pere B.S., Escayola C., Barri P.N. (2007). Ovulation induction treatment and risk of borderline ovarian tumors. Gynecol. Endocrinol..

[23] Sanner K., Conner P., Bergfeldt K., Dickman P., Sundfeldt K., Bergh T., Hagenfeldt K., Janson P.O., Nilsson S., Persson I. (2009). Ovarian epithelial neoplasia after hormonal infertility treatment: Long-term follow-up of a historical cohort in Sweden. Fertil. Steril..

[24] Van Leeuwen F.E., Klip H., Mooij T.M., van de Swaluw A.M., Lambalk C.B., Kortman M., Laven J.S., Jansen C.A., Helmerhorst F.M., Cohlen B.J. (2011). Risk of borderline and invasive ovarian tumours after ovarian stimulation for in vitro fertilization in a large Dutch cohort. Hum. Reprod..

[25] Yli-Kuha A.N., Gissler M., Klemetti R., Luoto R., Hemminki E. (2012). Cancer morbidity in a cohort of 9175 Finnish women treated for infertility. Hum. Reprod..

[26] Lundberg F.E., Johansson A.L.V., Rodriguez-Wallberg K., Gemzell-Danielsson K., Iliadou A.N. (2019). Assisted reproductive technology and risk of ovarian cancer and borderline tumors in parous women: A population-based cohort study. Eur. J. Epidemiol..

[27] Parazzini F., Negri E., La Vecchia C., Moroni S., Polatti A., Chiaffarino F., Surace M., Ricci E. (1998). Treatment for fertility and risk of ovarian tumors of borderline malignancy. Gynecol. Oncol..

[28] Huchon C., Bourdel N., Abdel Wahab C., Azais H., Bendifallah S., Bolze P.A., Brun J.L., Canlorbe G., Chauvet P., Chereau E. (2021). Borderline ovarian tumors: French guidelines from the CNGOF. Part 1. Epidemiology, biopathology, imaging and biomarkers. J. Gynecol. Obs. Hum. Reprod..

[29] Leake J.F., Currie J.L., Rosenshein N.B., Woodruff J.D. (1992). Long-term follow-up of serous ovarian tumors of low malignant potential. Gynecol. Oncol..

[30] Cadron I., Leunen K., Van Gorp T., Amant F., Neven P., Vergote I. (2007). Management of borderline ovarian neoplasms. J. Clin. Oncol..

[31] Rossing M.A., Daling J.R., Weiss N.S., Moore D.E., Self S.G. (1994). Ovarian tumors in a cohort of infertile women. N. Engl. J. Med..

[32] Buis C.C., van Leeuwen F.E., Mooij T.M., Burger C.W., Group O.P. (2013). Increased risk for ovarian cancer and borderline ovarian tumours in subfertile women with endometriosis. Hum. Reprod..

[33] Ness R.B., Cramer D.W., Goodman M.T., Kjaer S.K., Mallin K., Mosgaard B.J., Purdie D.M., Risch H.A., Vergona R., Wu A.H. (2002). Infertility, fertility drugs, and ovarian cancer: A pooled analysis of case-control studies. Am. J. Epidemiol..

[34] Harris R., Whittemore A.S., Itnyre J. (1992). Characteristics relating to ovarian cancer risk: Collaborative analysis of 12 US case-control studies. III. Epithelial tumors of low malignant potential in white women. Collaborative Ovarian Cancer Group. Am. J. Epidemiol..

[35] Barcroft J.F., Galazis N., Jones B.P., Getreu N., Bracewell-Milnes T., Grewal K.J., Sorbi F., Yazbek J., Lathouras K., Smith J.R. (2021). Fertility treatment and cancers-the eternal conundrum: A systematic review and meta-analysis. Hum. Reprod..

[36] Siristatidis C., Sergentanis T.N., Kanavidis P., Trivella M., Sotiraki M., Mavromatis I., Psaltopoulou T., Skalkidou A., Petridou E.T. (2013). Controlled ovarian hyperstimulation for IVF: Impact on ovarian, endometrial and cervical cancer—A systematic review and meta-analysis. Hum. Reprod. Update.

[37] Trope C.G., Kaern J., Davidson B. (2012). Borderline ovarian tumours. Best. Pract. Res. Clin. Obs. Gynaecol..

[38] Bourdel N., Huchon C., Abdel Wahab C., Azais H., Bendifallah S., Bolze P.A., Brun J.L., Canlorbe G., Chauvet P., Chereau E. (2021). Borderline ovarian tumors: Guidelines from the French national college of obstetricians and gynecologists (CNGOF). Eur. J. Obs. Gynecol. Reprod. Biol..

[39] Hauptmann S., Friedrich K., Redline R., Avril S. (2017). Ovarian borderline tumors in the 2014 WHO classification: Evolving concepts and diagnostic criteria. Virchows Arch..

