# Supplementary material for: Effects of Infertility Drug Exposure on the Risk of Borderline Ovarian Tumors: A Systematic Review and Meta-Analysis
Source: Biomedicines. 2023 Jun 26;11(7):1835. doi: 10.3390/biomedicines11071835 (PMC10376814; doi:10.3390/biomedicines11071835)
Supplement: Supplementary file 1 [file biomedicines-11-01835-s001.zip › Table S1-revised.pdf]

Supplementary Table S1. Newcastle–Ottawa Scale for assessment of quality of included studies—Case–control studies.

Quality assessment criteria	Acceptable(*)	Shushan et al. 1996 [20]	Parazzini et al. 1998 [27]	Cusidó et al. 2007 [22]
**Selection**				
Is the case definition adequate?	Yes, with independent validation	*	*	*
Representativeness of the cases?	Consecutive or obviously representative series of cases	*	*	*
Selection of controls?	Community controls	*	-	-
Definition of Controls?	No history of BOTs	*	*	*
**Comparability**				
Comparability of cases and controls on the basis of the design or analysis?	Study controls for the most important factor, Study controls for any additional factor	*	*	*
**Exposure**				
Ascertainment of exposure?	Secure record, Structured interview where blind to case/control status	-	-	*
Same method of ascertainment for cases and controls?	Yes	*	*	*
Non-Response Rate?	Same rate for both groups	*	*	-

Note: A study can be awarded a maximum of one star for each numbered item within the Selection and Exposure categories. A maximum of two stars can be given for Comparability.
