# Supplementary material for: Effects of Infertility Drug Exposure on the Risk of Borderline Ovarian Tumors: A Systematic Review and Meta-Analysis
Source: Biomedicines. 2023 Jun 26;11(7):1835. doi: 10.3390/biomedicines11071835 (PMC10376814; doi:10.3390/biomedicines11071835)
Supplement: Supplementary file 1 [file biomedicines-11-01835-s001.zip › Table S2-revised.pdf]

Supplementary Table S2. Newcastle–Ottawa Scale for assessment of quality of included studies—Cohort studies.

**Quality assessment criteria**	**Acceptable(*)**	**Sanner et al. 2009 [23]**	**Yli–Kuha et al. 2012 [25]**	**Stewart et al. 2013 [11]**	**Bjørnholt et al. 2015 [13]**	**Reigstad et al. 2017 [15]**	**Lundberg et al. 2019 [26]**	**Spaan et al. 2021 [14]**
**Selection**								
Representativeness of the exposed cohort?	Representative of the characteristics in the community	*	*	*	*	*	*	*
Selection of the non-exposed cohort?	Drawn from the same community as the exposed cohort	*	*	*	*	*	*	*
Ascertainment of exposure?	Secure record, structured interview	*	*	*	*	*	*	*
Demonstration that outcome of interest was not present at start of study?	Yes	*	*	*	*	*	*	*
**Comparability**								
Comparability of cohorts on the basis of the design or analysis?	Study controls for the most important factor, study controls for any additional factor	*	*	*	*	*	*	*
**Outcome**								
Assessment of outcome?	Independent blind assessment, record linkage	*	*	*	*	*	*	*
Was follow up long enough for outcomes to	Yes	*	*	*	*	*	*	*

occur?								
Adequacy of follow up of cohorts?	Complete follow up or a small number of subjects were lost to follow up but unlikely to introduce bias	*	-	-	*	-	*	*

Note: A study can be awarded a maximum of one star for each numbered item within the Selection and Outcome categories. A maximum of two stars can be given for Comparability.
