# Supplementary material for: Effects of Infertility Drug Exposure on the Risk of Borderline Ovarian Tumors: A Systematic Review and Meta-Analysis
Source: Biomedicines. 2023 Jun 26;11(7):1835. doi: 10.3390/biomedicines11071835 (PMC10376814; doi:10.3390/biomedicines11071835)
Supplement: Supplementary file 1 [file biomedicines-11-01835-s001.zip › Table S3-revised.pdf]

Supplementary Table S3. Counts for BOTs events and total events for exposures and controls.

Drugs of CC only.

Author, year	BOTs cases exposed to infertility drugs use	Total no. of infertility drugs use	BOTs cases not exposed to infertility drugs use	Total no. of non infertility drugs use
Shushan et al., 1996 [20]	2	20	34	424
Cusidó et al., 2007 [22]	4	20	38	279
Sanner et al., 2009 [23]	3	389	5	1,615
Bjørnholt et al., 2015 [13]	56	496	86	974
Reigstad et al., 2017 [15]	16	24,519	623	1,297,530
Spaan et al., 2021 [14]	13	6,515	28	9,712

Drugs of Gn only.

Author, year	BOTs cases exposed to infertility drugs use	Total no. of infertility drugs use	BOTs cases not exposed to infertility drugs use	Total no. of non infertility drugs use
Shushan et al., 1996 [20]	6	12	30	432
Cusidó et al., 2007 [22]	3	29	39	270
Sanner et al., 2009 [23]	1	325	5	1,615
Bjørnholt et al., 2015 [13]	22	278	87	1,159

Drugs of both (CC+Gn).

Author, year	BOTs cases	Total no. of	BOTs cases not exposed	Total no. of non

	exposed to infertility drugs use	infertility drugs use	to infertility drugs use	infertility drugs use
Shushan et al., 1996 [20]	8	32	28	412
Sanner et al., 2009 [23]	3	439	5	1,615

Infertility.

Author, year	BOTs cases exposed to infertility drugs use	Total no. of infertility drugs use	BOTs cases not exposed to infertility drugs use	Total no. of non infertility drugs use
Sanner et al., 2009 [23]	7	1,153	5	1,615
Stewart et al., 2013 [11]	17	7,544	14	14,095
Bjørnholt et al., 2015 [13]	89	772	53	698
Lundberg et al., 2019 [26]	27	25,208	39	49,208
Spaan et al., 2021 [14]	79	30,452	17	9,831

Nulliparous.

Author, year	BOTs cases exposed to infertility drugs use	Total no. of infertility drugs use	BOTs cases not exposed to infertility drugs use	Total no. of non infertility drugs use
Parazzini et al., 1998 [27]	2	2	35	136
Bjørnholt et al., 2015 [13]	50	277	32	224
Reigstad et al., 2017 [15]	12	14,645	245	584,338
Spaan et al., 2021 [14]	38	10,787	2	2,508

Parous.

Author, year	BOTs cases exposed to infertility drugs use	Total no. of infertility drugs use	BOTs cases not exposed to infertility drugs use	Total no. of non infertility drugs use
Parazzini et al., 1998 [17]	2	2	54	226
Bjørnholt et al., 2015 [13]	39	495	21	474
Reigstad et al., 2017 [15]	8	41,549	374	713,192
Lundberg et al., 2019 [26]	27	38,003	720	1,301,911
Spaan et al., 2021 [14]	41	19,665	15	7,323

Abbreviations: BOTs, borderline ovarian tumors; CC, clomiphene citrate; Gn, gonadotropin.
