# Supplementary figures and images for: Effects of Infertility Drug Exposure on the Risk of Borderline Ovarian Tumors: A Systematic Review and Meta-Analysis
Source: Biomedicines. 2023 Jun 26;11(7):1835. doi: 10.3390/biomedicines11071835 (PMC10376814; doi:10.3390/biomedicines11071835)

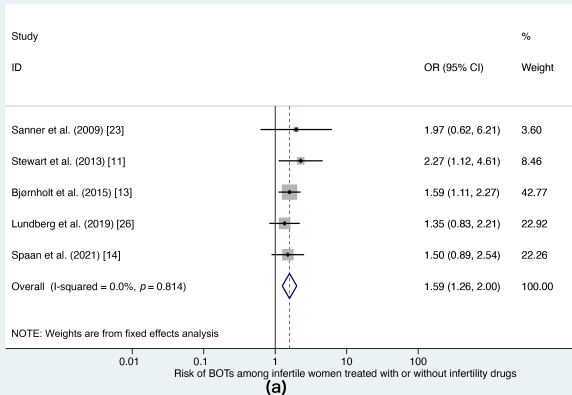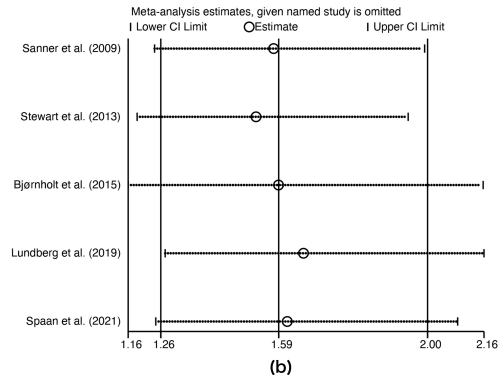

Supplement: Supplementary file 1 [file biomedicines-11-01835-s001.zip › Figure S1-revised.pdf]

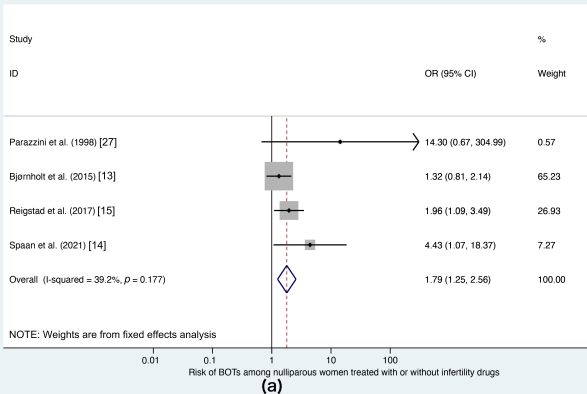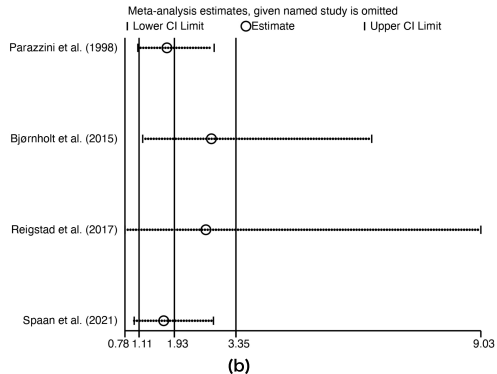

Supplement: Supplementary file 1 [file biomedicines-11-01835-s001.zip › Figure S2-revised.pdf]

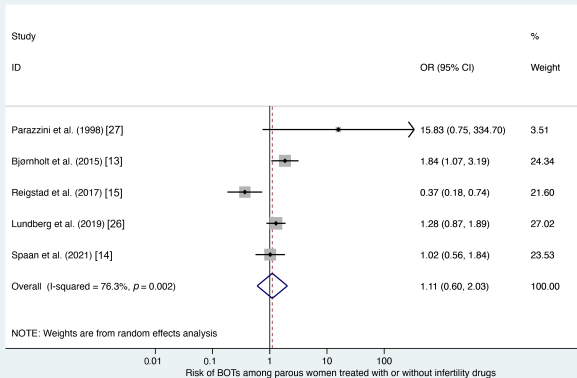

(a)

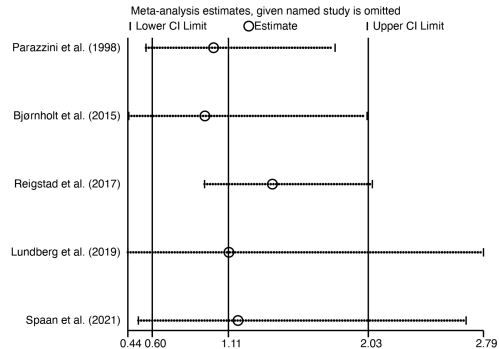

(b)

Supplement: Supplementary file 1 [file biomedicines-11-01835-s001.zip › Figure S3-revised.pdf]
